# Material Removal Characteristics of Abrasive-Free Cu Chemical-Mechanical Polishing (CMP) Using Electrolytic Ionization via Ni Electrodes

**DOI:** 10.3390/mi14020272

**Published:** 2023-01-20

**Authors:** Hyunseop Lee

**Affiliations:** Department of Mechanical Engineering, Dong-A University, Busan 49315, Republic of Korea; hyunseop@dau.ac.kr; Tel.: +82-51-200-7648

**Keywords:** chemical mechanical polishing (CMP), abrasive-free CMP, electrolytic ionization, copper, nickel electrode

## Abstract

Recently, various efforts have been made to reduce the environmental burden caused by semiconductor manufacturing by improving the process efficiency. Chemical mechanical polishing (CMP), which is used to planarize thin films in semiconductor production, has also been studied to improve its efficiency by increasing the material removal rate (MRR) while reducing its environmental burden. Previous studies have been conducted to electrolytically ionize chemical solutions used in abrasive-free CMP for improving the MRR. In this study, we analyzed the change in the chemical solution according to the variation in voltage applied to the nickel (Ni) electrode in abrasive-free Cu CMP and studied the tribological material removal characteristics. The experimental results revealed that electrolytic ionization of the chemical solution for abrasive-free CMP increases the amount of dissolved oxygen (DO). The static etch rate of the Cu thin film and MRR in CMP increased as the voltage applied to the Ni electrode increased. The frictional force and temperature during CMP also increased as the applied voltage increased. Therefore, the increase in MRR caused by the increase in the applied voltage in abrasive-free Cu CMP using electrolytic ionization is plausibly caused by the chemical reaction between the dissolved oxygen in the chemical solution and Cu.

## 1. Introduction

With the high integration of semiconductors, chemical mechanical polishing (CMP) technology has developed remarkably, and its application scope is gradually expanding [1,2,3]. Recently, CMP technology has been applied not only to the planarization of various thin-film materials but also to the processing of new substrate materials. The development of CMP technology has contributed significantly to the development of displays, electronic components, and semiconductors. However, because CMP uses chemical solutions and abrasive particles, it poses an environmental burden and leads to defects such as scratches, dishing, and erosion [4,5]. In particular, polished materials are at a risk of developing scratches owing to the agglomeration of abrasive particles in the CMP chemical solution. Several studies have attempted to solve CMP problems.

Some researchers have proposed a method using an abrasive-free slurry for resolving defects that may be caused by abrasive particles in CMP. Abrasive-free CMP has been studied for planarizing a Cu film because the chemically reactive layer on the surface of Cu can be removed by the asperities of the polishing pad by adjusting the chemical composition of the abrasive particle-free slurry. Hanazono et al. [6] reported that abrasive-free Cu CMP involves a material removal mechanism in which a Cu-complex layer generated via a chemical reaction is removed using a pad. In addition, because of the absence of abrasive particles in the slurry, post-treatment of the waste abrasive particles after CMP is not required. Pandija et al. [7] introduced an abrasive-free Cu CMP chemical solution containing oxalic acid and hydrogen peroxide (H_2_O_2_) and showed that the material removal rate (MRR) could be controlled by adjusting the content and pH. Oxalic acid and H_2_O_2_ were used as complexing and oxidizing agents, respectively. Their study demonstrated the highest MRR in an abrasive-free Cu CMP solution with pH of 3–4. Ramakrishnan et al. [8] used dicarboxylic acids (oxalic, malonic, succinic, and glutaric acids) as the complexing agent of the chemical solution and showed that a suitable complexing agent should be selected according to the pH of the chemical solution. The mechanical material removal in abrasive-free CMP is only caused by the relative motion of the polishing pad asperities and wafers; thus, the interfacial friction characteristics are related to material removal. DeNardis et al. [9] stated that Cu-abrasive-free CMP is similar to the friction behavior of interlayer dielectric (ILD) CMP and that the generation cycle of the Cu complex layer is approximately 10 ms. Haque et al. [10] proposed an MRR model that considers the contact area between the asperities of the polishing pad and wafer in abrasive-free CMP and the etching of copper under low- and high-pressure conditions. Thus, when the mechanical factors are fixed, the MRR of abrasive-free CMP depends on the degree of chemical activation of the chemical solution. Accordingly, various methods for increasing the chemical activity of slurry in the CMP process have been studied.

DeNardis et al. [11,12] conducted studies to control the amount of oxygen supplied to a chamber using a controlled atmosphere polishing (CAP) system and revealed that the MRR in Cu CMP increased as the amount of dissolved oxygen (DO) increased. Increasing the temperature of the slurry is one way of increasing chemical activity. During the CMP process, the temperature of the polishing pad increases owing to the heat generated by friction, which also affects the MRR [13]. Sorooshian et al. [14] studied the Arrhenius characteristics in ILD and Cu CMPs and revealed that Cu CMP has a higher combined activation energy than ILD CMP. Mudhivarthi et al. [15] experimentally demonstrated that the MRR and coefficient of friction (CoF) increase as the interfacial temperature increases in Cu CMP. 

Another method for improving the MRR of CMP is the electrolytic ionization of the slurry by applying electrical energy. Lee et al. [16] conducted a study to improve the MRR in Cu CMP by electrolytically ionizing the CMP slurry. They used a DC voltage for ionization and stainless steel as the electrode. They confirmed that particle aggregation occurred when using a colloidal silica slurry and fumed silica slurry was used for the experiment. In addition, surface chemical analysis revealed that the electrolytically ionized slurry generated more oxidized copper than the general slurry, and using UV-vis spectroscopy, the electrolytic ionized slurry was confirmed to generate more ·OH radicals. Based on the research by Lee et al. [16], Park and Lee [17] introduced electrolytically ionized abrasive-free Cu CMP. In their study, stainless steel was used as the electrode material, and an AC power supply was used to apply a voltage (30 V). The results of the general abrasive-free CMP and abrasive-free CMP using electrolytic ionization were compared. In addition, the general abrasive-free CMP and abrasive-free CMP using electrolytic ionization were compared.

Existing CMP studies that use slurry electrolytic ionization primarily use stainless steel as an electrode material. The change in the chemical solution through electrolytic ionization has not been investigated. In this study, nickel (Ni), exhibiting higher conductivity than stainless steel, was used as an electrode material, and the change in the chemical solution used in abrasive-free Cu CMP, depending on electrolytic ionization, was studied. A tribological study was conducted on the MRR, friction force, and pad temperature changes based on the voltage applied during the abrasive-free CMP using electrolytic ionization. In addition, to investigate the material removal characteristics of abrasive-free Cu CMP using electrolytic ionization, the amount of dissolved oxygen in the slurry was measured, unlike in previous studies.

## 2. Experimental Conditions

**Figure 1:**
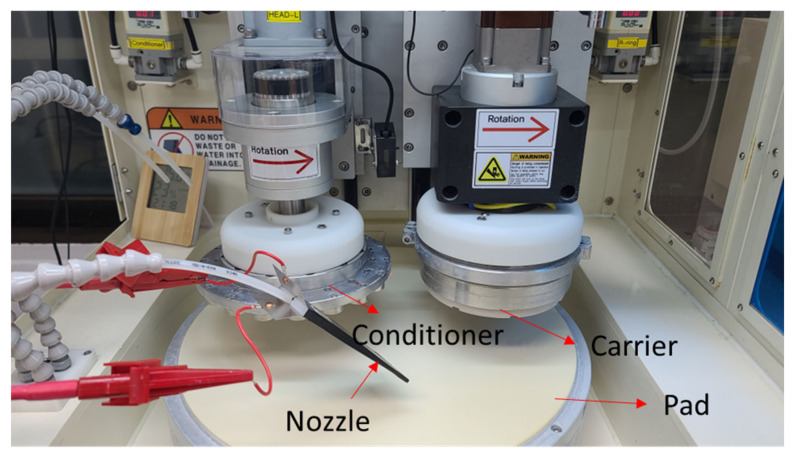
CMP system (POLI-400, GnP Technology Inc., Busan, Korea).

**Figure 2:**
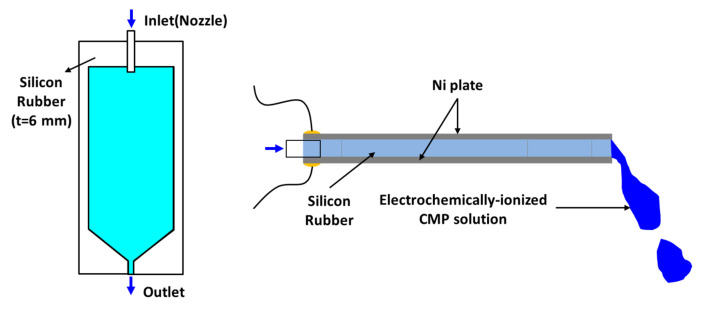
Schematic of the slurry supply system.

A CMP system for R&D (POLI-400, GnP Technology Inc., Busan, Korea) was used, and an electrolytic ionization device for the chemical solution using Ni plates was placed at the end of the nozzle (Figure 1). The dimensions of the Ni plate were 45 × 150 × 0.35 mm, and two Ni plates were arranged as illustrated in Figure 2. The chemical solution flowed between the two plates and was supplied to the polishing pad. An AC power supply (APS-7100E(CE), GW Instek, New Taipei City, Taiwan) was used to supply voltage of 0–30 V to the electrodes. The frequency was set at 60 Hz.

The CMP solutions used in the experiment included deionized water (DIW), H_2_O_2_, citric acid, and benzotriazole (BTA). H_2_O_2_, citric acid, and BTA act as an oxidizer, complexing agent, and corrosion inhibitor, respectively. The concentration of each component of the CMP solution is listed in Table 1. The pH of the CMP solution was ~3.7.

The change in thickness of the Cu thin film was measured using a 4-point probe (CMT-100S, AiT Co., Suwon, Korea). The CMP MRR was expressed as the change in the thickness of the Cu thin film removed for 1 min, and the within-wafer non-uniformity (WIWNU) was the percentage obtained by dividing the standard deviation (STD) of the MRR, measured at 19 points in the wafer radius direction, by the average MRR.

A square coupon wafer (30 × 30 mm) was immersed in an electrolytic ionized chemical solution for 5 min and the static etch rate of the Cu thin film was measured. Surface chemical analysis was performed using scanning electron microscopy (SEM) and X-ray photoelectron spectroscopy (XPS) (AXIS SUPRA, KRATOS Analytical Ltd., Manchester, UK). The change in thickness of the Cu thin film was measured at five points on the coupon wafer. To confirm the change in the electrolytically ionized CMP solution, the amount of dissolved oxygen after electrolytic ionization was measured.

In the CMP experiment, 4-inch wafers were used and 1 μm of sputtered Cu was deposited on the Ti barrier (50 nm). A hard polyurethane polishing pad (KPX Chemical, Seoul, Republic of Korea) was used. The CMP experimental conditions are listed in Table 2. The wafer and retainer pressures were 27.46 kPa (4 psi) and 37.27 kPa (5 psi), respectively, and the rotation speed of the platen and head was 80 rpm. The distance between the centers of the polishing head and pad was fixed at 130 mm. The flow rate of the chemical solution was set at 150 mL/min. During the CMP experiment, the friction force was measured using a force sensor, and the temperature change at the trailing edge of the wafer was measured using a portable infrared thermometer (GM-320, BENETECH, Shenzhen, China), respectively.

## 3. Results and Discussion

**Figure 3:**
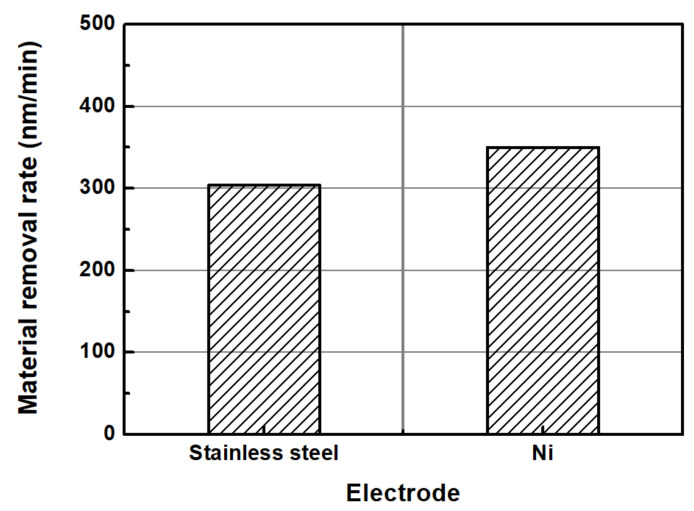
Comparison of the MRR by electrode type (SUS vs. Ni, applied voltage: 30 V).

Figure 3 illustrates the MRRs when an abrasive-free CMP solution was electrolytically ionized at 30 V using the stainless steel and Ni electrodes. In accordance with Park et al. [17], the MRR was 303.9 nm/min when a voltage of 30 V was applied to the abrasive-free CMP solution using the SUS electrode. Ni is known to have a higher conductivity than SUS; therefore, electrolytically ionizing an abrasive-free CMP solution more efficiently seems possible. When Ni electrodes were used under the same processing conditions, the MRR increased by approximately 13.2%.

**Figure 4:**
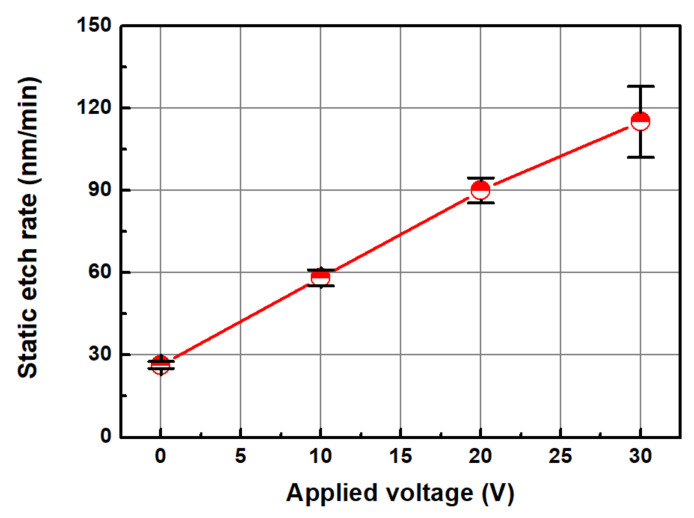
Static etch rate as a function of the applied voltage.

**Figure 5:**
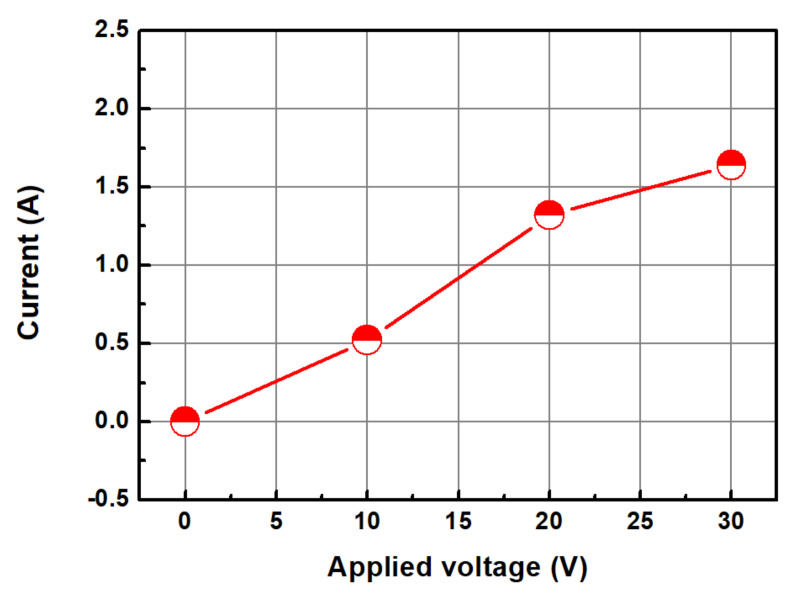
Change in current according to the voltage applied to the electrode.

Figure 4 shows the change in the static etch rate as a function of the applied voltage. To measure the static etch rate, a coupon wafer was placed in a beaker, a slurry passing through the Ni electrode was supplied to the container for 3 min, and the change in thickness of the Cu film was measured. The static etch rate was measured using a four-point probe for determining the change in the thickness of the Cu thin film at five points on a square coupon wafer (30 mm × 30 mm). Figure 5 shows the changes in current with an increase in the voltage applied to the electrode. As the voltage increased, the current increased linearly. A general abrasive-free CMP condition was achieved when the applied voltage was 0 V and the static etch rate was 26.4 nm/min. As the applied voltage increased, the static etch rate of the copper increased. The static etch rates at the applied voltages of 10, 20, and 30 V were 58.2, 90.0, and 115.0 m/min, respectively. 

**Figure 6:**
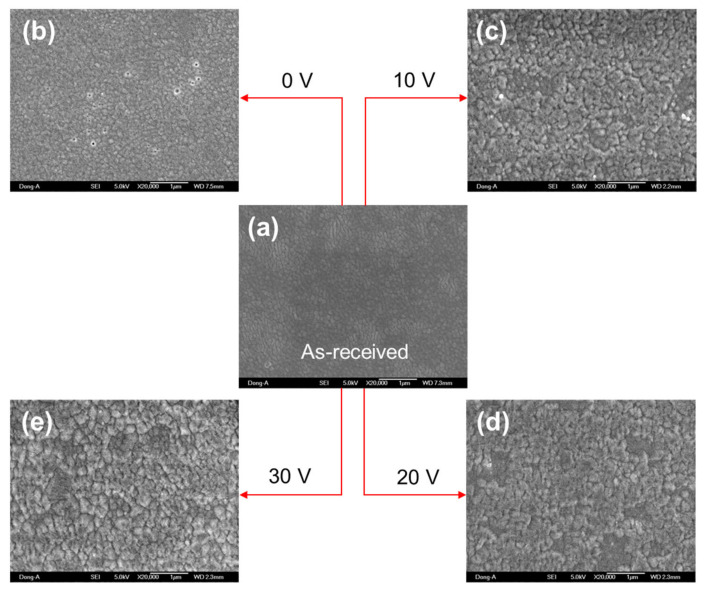
SEM images (×20,000) of the etched surface of Cu; (**a**) as-received, (**b**) 0 V, (**c**) 10 V, (**d**) 20 V, and (**e**) 30 V.

Figure 6 shows the SEM images of the etched wafers. Figure 6a shows a surface image of the as-received (as-deposited) wafer. Figure 6b–d show the surface of the etched copper at voltages of 10, 20, and 30 V, respectively, on the Ni electrode. The SEM images show that the surface of the Cu wafer immersed in the electrolytic ionized solution was rougher and more oxidized than that of the as-received wafer. The change in the amount of static etching due to the increase in the applied voltage is associated with the MRR in CMP and produces a chemically reactive layer that is mechanically easy to remove from the surface of Cu.

**Figure 7:**
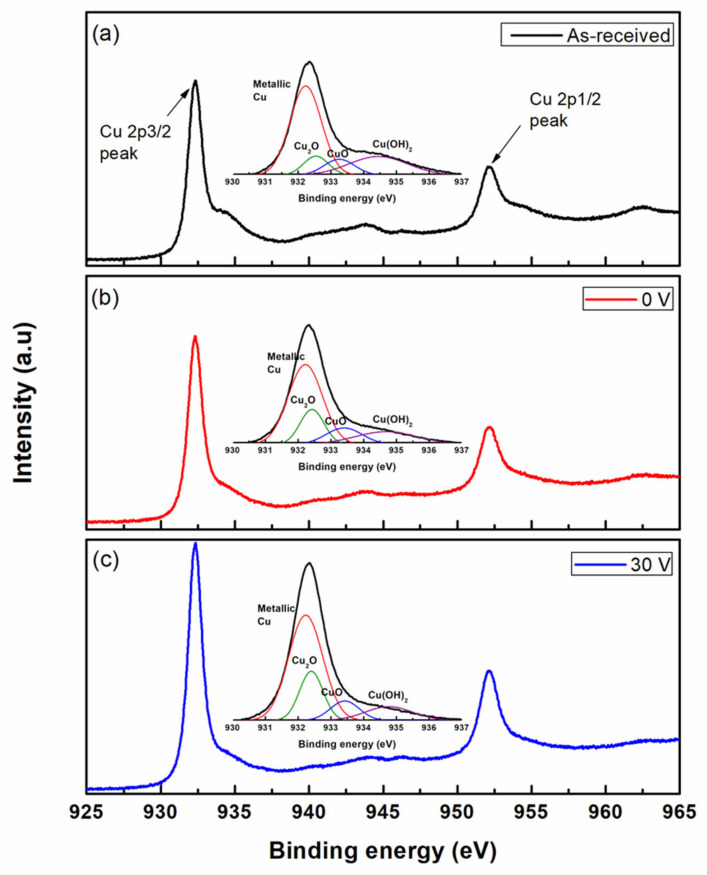
XPS spectra of the as-received Cu wafer; (**a**) as-received, (**b**) 0 V, and (**c**) 30 V.

Figure 7 shows the results obtained by analyzing the wafer surface after the etching experiment. Figure 7a shows the results of the analysis of the Cu thin films before the experiment. The Cu 2p spectra were divided into the Cu 2p1/2 and Cu 2p3/2 peaks. Cu is easily oxidized in the atmosphere, and a native oxide film forms on its surface. Native copper oxide exists mainly in the form of cuprous oxide (Cu_2_O), cupric oxide (CuO), and cupric hydroxide (Cu(OH)_2_). The binding energies of metallic Cu 2p1/2 and Cu 2p3/2 were approximately 952.3 and 932.6 eV, respectively. The binding energies of metallic Cu, CuO, Cu(OH)_2_, and Cu_2_O have been reported to be approximately 932.6 [18], 933.5 ± 0.2 [18,19,20], 934.5 ± 0.2 [19], and 932.18 ± 0.12 eV [18], respectively. At 0 V (Figure 7b), The intensity of Cu(OH)_2_ was lower than that of the as-received Cu, whereas an increase in the intensity of the Cu_2_O and CuO peaks was observed. At 30 V (Figure 7c), the Cu_2_O and CuO peaks were higher than those of the as-received Cu and those at 0 V. According to the Cu CMP mechanism, the Cu surface is oxidized by the oxidizer in the slurry and Cu ions are generated, and the etching of Cu occurs by combining the complexing agent and generated Cu ions. Figure 7 shows that oxidation of the Cu surface was more active than that in a general abrasive-free Cu CMP when the chemical solution was electrolytically ionized using a Ni electrode.

**Figure 8:**
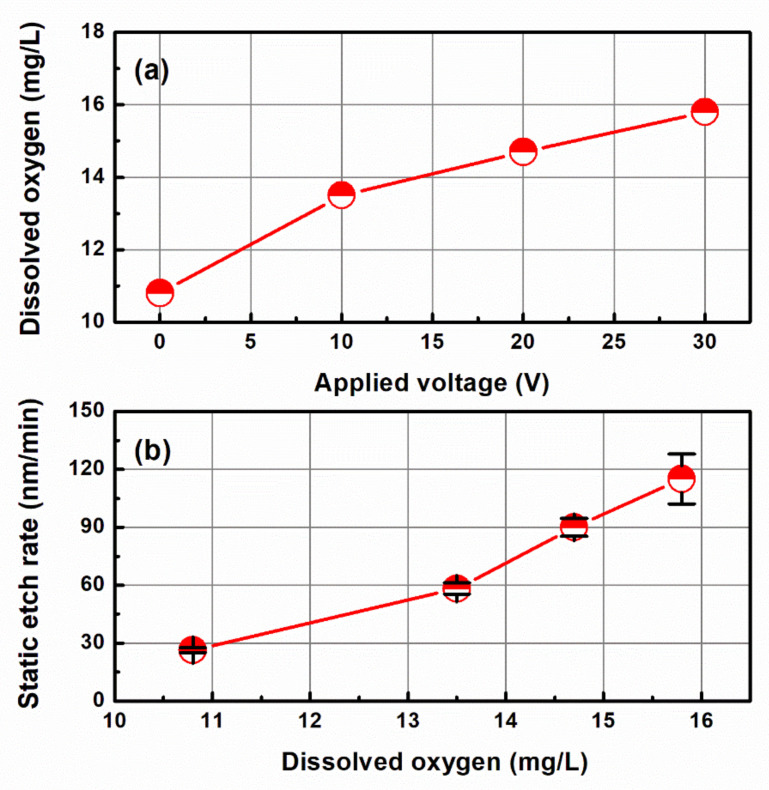
Dissolved oxygen in the chemical solution as a function of the applied voltage (**a**) and the static etch rate as a function of the dissolved oxygen (**b**).

Previous studies on CMP using electrolytic ionization explained the CMP mechanism through the analysis of chemically reacted surfaces, but the change in dissolved oxygen due to electrolytic ionization was not considered. Figure 8a shows the change in the dissolved oxygen in the chemical solution with respect to the applied voltage. When the applied voltage was 0 V, the dissolved oxygen was 10.8 mg/L, and at 10, 20, and 30 V, the dissolved oxygen concentrations were 13.5, 14.7, and 15.8 mg/L, respectively. This appears to be because DIW, which corresponds to the base chemical of the chemical solution, generated oxygen via electrolysis, as shown in Equation (1).

(1) $$2H_{2}O\;\to 2H_{2}+O_{2}$$

Imai et al. [21] investigated the effect of dissolved oxygen in DIW on the corrosion of Cu during the wafer-cleaning process and revealed that the etch rate of Cu increased as the dissolved oxygen increased. They reported that the Cu surface was ionized by DIW with a high dissolved-oxygen content. The oxidation mechanism of copper by dissolved oxygen is as follows [21]:

(2) $$Cu+\frac{1}{2}O_{2}\;\to CuO$$

(3) $$CuO+2H^{+}\;\to \;Cu_{2}^{+}+H_{2}O$$

Figure 8b illustrates the change in the static etch rate according to the dissolved oxygen. As in the results of Imai et al. [21], the static etch rate of Cu tends to increase as the dissolved oxygen in the chemical solution increases. DeNardis et al. [11,12] experimentally demonstrated that the MRR can be increased by creating an O_2_ gas atmosphere during Cu CMP. Jo et al. [22] used an atomization system for improving the MRR by supplying slurry with O_2_ gas. The CMP method, which uses electrolytic ionization, increases the amount of dissolved oxygen through electrolytic ionization of a chemical solution instead of directly injecting O_2_ gas.

**Figure 9:**
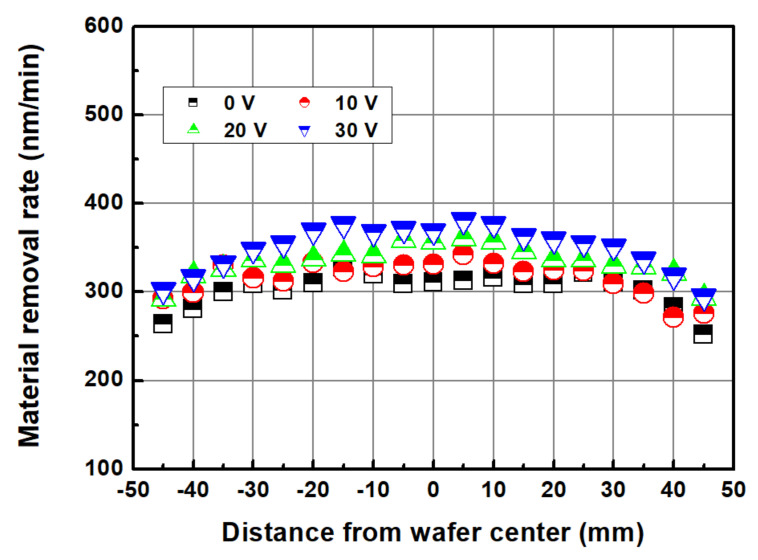
Material removal rate distribution according to the applied voltage.

**Figure 10:**
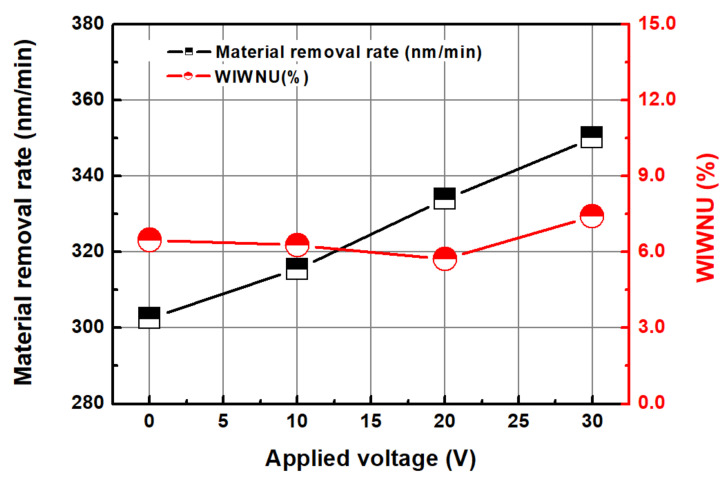
Average material removal rate and WIWNU as a function of the applied voltage.

Figure 9 shows the MRR distribution (edge exclusion (EE) of 5 mm) according to the applied voltage in the abrasive-free Cu CMP using an electrolytically ionized chemical solution. The output currents at the applied voltages of 10, 20, and 30 V were 0.52, 1.32, and 1.64 A, respectively. As shown in Figure 10, the average MRR tended to increase with an increase in the applied voltage. At the applied voltages of 0, 10, 20, and 30 V, the MRRs were 302.5, 315.5, 333.9, and 350.1 nm/min, respectively. The WIWNU values were 6.47, 6.27, 5.72, and 7.41% at 0, 10, 20, and 30 V, respectively. The WIWNU were not significantly different depending on the conditions but showed a slight deterioration at 30 V. As shown in Figure 9, the MRR profile generally exhibits a high and low MRR at the center of the wafer and near the edge, respectively. In CMP, the MRR distribution is known to follow pressure (stress), relative velocity, and chemical reaction distributions. The chemical reaction distribution is related to the sliding distance and temperature distribution. In this study, the relative speed of the wafer to the polishing pad was constant at 1.09 m/s (rotational speed of the platen and head was 80 rpm) at any position of the wafer. The sliding distance distribution was also constant because the distance between the centers of the polishing head and pad was fixed, and the rotational speed of the wafer and polishing pad was the same. The pressure acting on the wafer was the same in all experiments. Therefore, the changes in the MRR and MRR profiles in Figure 9 and Figure 10 appear to be a result of the change in chemical reactivity according to the electrolytic ionization of the chemical solution using the Ni electrode.

**Figure 11:**
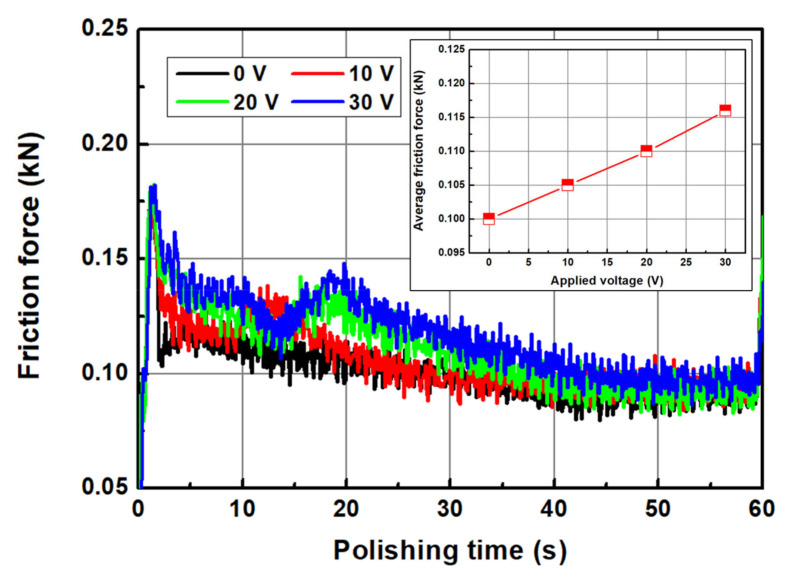
Friction force according to the applied voltage and the average friction force as a function of the applied voltage.

**Figure 12:**
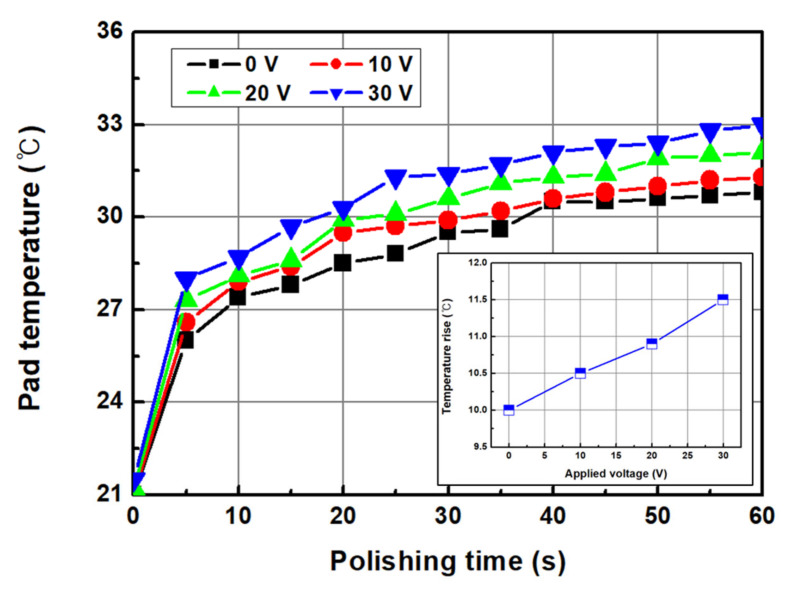
Pad temperature according to the applied voltage and average pad temperature as a function of the applied voltage.

The friction force (Figure 11) and polishing-pad temperature (Figure 12) at the trailing edge of the wafer were measured during CMP. When no voltage was applied to the electrode (0 V), the friction force in CMP tended to decrease continuously. However, when a voltage was applied, the frictional force decreased at the beginning of the processing and then increased and decreased again. The reduction in the frictional force in the early stages of processing is attributed to the removal of the native oxide layer on the wafer surface. The average frictional forces were 0.100, 0.105, 0.110, and 0.116 kN at 0, 10, 20, and 30 V, respectively, and the frictional force increased as the applied voltage increased. Compared to general abrasive-free CMP, the friction force in abrasive-free CMP that uses an electrolyzed chemical solution tends to be similar to that obtained by Park and Lee [17].

In the CMP process, directly measuring the frictional heat generated at the interface between the wafer and pad is difficult. However, it can be identified indirectly by measuring the pad temperature near the trailing edge of the wafer. The average temperature of the polishing pad before CMP was 21.2 °C, and as CMP progressed, the pad temperature near the trailing edge of the wafer continued to increase. The average temperature of the polishing pad tended to increase as the applied voltage increased, and the average temperatures were 28.6, 29.1, 29.7, and 30.4 °C for 0, 10, 20, and 30 V, respectively. The increases in the polishing pad temperature were 10.0, 10.5, 10.9, and 11.5 °C at 0, 10, 20, and 30 V, respectively. The increase in the friction force with increasing applied voltage was attributed to the formation of a chemically reactive layer owing to the increase in dissolved oxygen in the chemical solution. The polishing pad temperature is thought to be a result of the interfacial friction between the polishing pad and wafer. An increase in process temperature during CMP promotes the chemical reaction of the slurry.

As a result of observing the change in the chemical solution for abrasive-free Cu CMP and analyzing the CMP experiment results according to the increase in the applied voltage, electrolytic ionization of the chemical solution seems to improve the chemical reactivity by increasing the dissolved oxygen in the solution. The chemically reactive layer on the copper surface generated by the activation of the chemical solution appears to be removed by mechanical friction owing to the asperities of the polishing pad.

## 4. Conclusions

In this study, abrasive-free Cu CMP using electrolytic ionization with a Ni electrode was investigated. A change in the chemical solution was observed with an increase in the applied voltage. In this study, changes in the amount of dissolved oxygen and the chemical composition of Cu according to the electrolytic ionization of the chemical solution were investigated. When electrolytic ionization of the chemical solution was achieved, the dissolved oxygen in the chemical solution increased through electrolytic ionization. The increased dissolved oxygen in the chemical solution promotes a chemical reaction with Cu, increasing the static etch rate of Cu and leaving a chemical reaction layer. As the voltage applied to the electrode increased, the MRR of Cu, frictional force, and temperature during processing also increased. This result appears to be because a reactive layer that is easily removed mechanically is formed on the surface of Cu through electrolytic ionization of the chemical solution, and as the applied voltage increases, the asperities of the polishing pad remove the reactive layer.

Although an Ni electrode was used in this study, more research on various electrode materials is needed in the future, and the design and optimization of a nozzle for electrolytic ionization are required. In addition, studies applying the CMP method using electrolytic ionization to the planarization of various materials that require longer processing times should be conducted.

## Figures and Tables

**Table 1:** Chemical composition of abrasive-free CMP solution.

Chemical	Concentration (wt%)
Deionized water (DIW)	96.75
Hydrogen peroxide (H_2_O_2_)	3.00
Citric acid	0.20
Benzotriazole (BTA)	0.05

**Table 2:** Abrasive-free CMP process condition.

Parameter	Value
Wafer pressure (kPa)	24.76
Retainer pressure (kPa)	37.27
Head oscillation	No
Platen speed (rpm)	80
Head speed (rpm)	80
Flow rate (mL/min)	150
Applied voltage (V)	0, 10, 20, 30
Polishing time (s)	60

## Data Availability

Not applicable.
